# Investigating men’s motivations to engage in genetic screening for *BRCA1* and *BRCA2* mutations

**DOI:** 10.1371/journal.pone.0265387

**Published:** 2022-03-18

**Authors:** Anna Maria Annoni, Claudia Longhini

**Affiliations:** 1 Institute of Public Health, Faculty of Biomedical Sciences, Università della Svizzera Italiana, Lugano, Switzerland; 2 Department of Business Economics, Competence Centre on Ageing, Health and Social Care, University of Applied Sciences and Arts of Southern Switzerland, Manno, Switzerland; The Ohio State University, UNITED STATES

## Abstract

*BRCA1* and *BRCA2* mutations are associated with an increased risk of developing numerous cancers, including breast, ovarian, pancreatic, melanoma and prostate cancer. Men face *BRCA*-related cancer risks as women do. However, there is considerably less research on the psychological determinants of men engaging in *BRCA1/2-*related cancer prevention compared to women. The present research aimed to study the determinants of men’s motivations to engage in genetic screening for *BRCA1* and *BRCA2* through the lens of the Health Action Process Approach. One hundred and twenty-five men (mean age = 58.53 y/o, SD = 10.37) completed an online survey. The intention to undergo genetic screening for *BRCA1/2* mutations in men was significantly and positively associated with self-efficacy and risk perception. Moreover, having offspring positively affected intention as well. The relationships between intention (and planning) and positive outcome expectancies, age, and family history of breast-related cancer were not statistically significant. Most information on *BRCA1* and *BRCA2* mutations is tailored to women due to the availability of effective surgical risk reduction procedures for women’s breast and ovarian cancer. Future research should focus on the best methods of communicating informed decision-making for men facing the risk of such mutations.

## Introduction

*BRCA1-* and *BRCA2-*associated Hereditary Breast and Ovarian Cancer Syndrome (HBOC) increases the risk of developing breast, ovarian cancer, as well as prostate cancer, pancreatic cancer and melanoma [1–6].

The prevalence of *BRCA1* and *BRCA2* mutations in the general population is around 0.2% (1/500), with wide variations by country [7] or context [5], but both sexes can present with mutations. *BRCA1* and *BRCA2* pathogenic mutations are transmitted in an autosomal dominant mode, meaning that the probability of being a carrier is 50% with a mutation in a first-degree relative (parent, brother/sister, son/daughter [5, 8]). The heritability is associated with an increased likelihood of clinically significant *BRCA* mutations [9]. Approximately 10% of men with breast cancer are genetically predisposed, which, in most cases, is determined by hereditary *BRCA1* and *BRCA2* mutations [10].

In the general male population, the lifetime risk of developing breast cancer is 0.1%; whereas in *BRCA1* mutations it is 1.2% by the age of 70, rising to 6.8% in the case of *BRCA2* [5]. A meta-analysis reported a moderate association between *BRCA1* mutation and prostate cancer [11]. The risk of prostate cancer by the age of 69 is 6%, while with *BRCA1* mutations, it rises to 8.6% by the age of 65. In the case of *BRCA2* mutations, the risk of developing prostate cancer by 65 is 15% [5, 12]. Approximately 20% of men diagnosed with breast cancer present a family history of breast cancer in first-degree relatives, 2% develop secondary breast cancer, and more than 20% develop a second non-breast cancer (mainly prostate cancer [13, 14]).

The available information on *BRCA1/2*-related cancers is directed mainly at women, reflecting a gendered approach that may lead men to underestimate their risk of carrying *BRCA* mutations [15]. Moreover, while a good proportion of women’s *BRCA*-related breast cancers are detected with screening, in men, the detection follows the onset of symptoms, and often at later stages [16, 17]. In the U.S., the ratio of female to male testing for *BRCA1* and *BRCA2* mutations exceeds 10:1 [18, 19]. There is a lot of research on the psychological determinants of the motivations of women to engage in cancer-preventive behaviors [20, 21] and specifically in *BRCA1-* and *BRCA2-*related cancer prevention [22–24], but there is still not enough information on men’s motivations. Available evidence suggests that passive avoidance of risk management is common among men. Although they appear to be open to receiving information on genetic mutations and their consequences for health, they are less likely to actively seek, test and screen for *BRCA1/2*-related cancer risks, underestimating the likelihood of developing cancer [25, 26]. Furthermore, Rauscher et al. (2019) [27] found that men were prone to show passive attitudes towards screening, and were less likely to seek information and take action to manage their risk. Additionally, men have different patterns and coping strategies than women in families; which means men are less likely to be included in cancer risk conversations and less likely to initiate preventive actions [25, 28, 29]. Risk awareness on *BRCA*-related mutations and cancers transmission is conveyed using gender-specific communication and psychological approaches. Consequently, men are more likely than women to be disorientated about risk information or recognize themselves as targeted by sensibilization campaigns. This leads to the common misbelief of considering *BRCA* screening recommendations as targeted on women [19], which strengthens insufficient or incorrect knowledge on risks and risk management among men.

The lack of information tailored to men’s specific needs regarding *BRCA 1/2-*related cancer risks, and the limited and less known options available for prevention and treatments, make men’s *BRCA*-related cancer management uncertain and in need of further study. Nevertheless, researchers exploring the intentionality of men in approaching genetic screening and testing have done so through qualitative studies [27, 28] or in clinical samples [30, 31]. This calls for a better understanding of the antecedents of preventive behavior. The present research aimed to understand the determinants of men’s motivations towards in genetic screening for *BRCA1* and *BRCA2* through the application of principles of the Health Action Process Approach.

### The health action process approach

Health self-regulation is the motivational, volitional, and behavioral process promoting the replacement of health-compromising behaviors with health-enhancing behaviors [32]. The Health Action Process Approach (HAPA) is a theoretical model developed by Schwarzer (2008) [33] that seeks to understand the distal and proximal determinants of behavioral change.

The pre-intentional motivational phase includes the distal antecedents of the formation of the intention to act: risk perception, positive outcome expectancies, and a re-elaborated role of Bandura’s [34, 35] self-efficacy theory in enhancing health behaviors [36]. Risk perception is the subjective evaluation that an individual makes about the probability and the severity of developing a specific disease. This evaluation is a booster for motivation [37] and promotes preventive behaviors [38]. Positive outcome expectancies consist of the expected social, physical, and emotional consequences of the behavioral enhancing [33]. They consist of the perceived advantages associated with behavioral change, reflecting the positive consequences for an individual undergoing genetic screening (e.g., being appreciated by other family members for the effort of will, updated information on health status). Finally, self-efficacy refers to the belief in the ability to succeed and is considered the core feature of the social cognitive theory of Bandura [34, 35]. Self-efficacy involves all the past experiences, motivations, affective states, and interests required to successfully perform a specific task, strongly predicting a wide range of short- and long-term health-related behaviors [39–42]. Self-efficacy also plays a critical role in the decision to undergo genetic screening and face the predicted consequences [43, 44], as the role played in *BRCA1/2* mutation testing in women [45, 46]. Risk perception, positive outcome expectancies and self-efficacy constitute the motivational phases and reflect the operational definitions of social-cognitive predictors of the intention to enhance a specific health behavior, and the planning of the requisite concrete steps.

The HAPA model has been applied to understand the intention to take up cervical cancer screening [47], as well as dietary behavior and physical activity among coronary and hypertensive patients [48, 49]. The present research intends to apply principles from the HAPA model to predict men’s intention to undergo genetic screening, controlling for the presence of offspring and family history of *BRCA1/2* related cancers, which is related to higher risk of *BRCA1* and *BRCA2* mutations.

## Method

### Procedure

Participants were recruited through snowball sampling and social networks advertising, targeting adult males (18+) who were fluent in Italian. In addition, the audience was targeted to reach stakeholders in well-known local not-for-profit cancer research foundations. Exclusion criteria included the presence of ascertained genetic *BRCA1*/2 mutations and/or a cancer diagnosis. The study received approval from the Institutional Review Board of the Università della Svizzera italiana. Participation was voluntary and no monetary compensation was provided. All measures were self-reported and anonymous, and both informed consent and data were collected through Qualtrics, an online survey platform. Informed consent was obtained as part of the Qualtrics survey.

### Measures

#### Family history of BRCA1/2 cancer

An adapted version of the Seven question Family History Screening (FHS-7 [50]) that is suitable for men and women was administered to collect information about family history of breast, ovarian and prostatic cancer. The questions were as follows: (1) "Have any of your first-degree relatives been diagnosed with breast or ovarian cancer?"; (2) "Have any of your relatives been diagnosed with bilateral breast cancer?"; (3) "Have any man in your family ever been diagnosed with breast and/or prostatic cancer?"; (4) "Have any woman in your family been diagnosed with breast and/or ovarian cancer?"; (5) "Have any woman in your family been diagnosed breast cancer before the age of 50?"; (6) "Do you have two or more relatives with breast and/or ovarian cancer?"; (7) "Do you have two or more relatives with breast and/or prostatic cancer?". Response categories included "Yes", "No", and "I do not know". According to Ashton-Prolla et al. [50], participants who reported at least one positive answer were considered at risk for *BRCA* mutations.

#### Risk perception

Risk perception was assessed with one question that focused on relative health risk [51, 52]: "Compared to people similar to you in age and gender, your chances of having prostate and/or breast cancer in the future are …". Respondents answered using a 7-point Likert scale that ranged from 1 "far below average" to 7 "greater than above average". (M = 3.56, SD = 1.38).

#### Positive outcome expectancies

The following five questions were adapted from previous literature [33, 53, 54] assessing the extent of positive outcome expectancies of genetic screening: "In doing a genetic screening, how likely are each of the following scenarios: "It would increase my sense of security"; "Other people and my family members would appreciate the effort of will"; "I would be proud to take care of myself"; "It would be good for my family members and me"; and "I would have important information for my health". The response scale was a 5-point Likert scale ranging from 1 “unlikely” to 5 “very likely”. (M = 4.02, SD = 0.92, α = .852, *r* = .550).

#### Self-efficacy

Consistent with Schwarzer (2008) [33], self-efficacy was assessed by one’s capability of keeping up with the behavior and by implementing coping strategies: "Now indicate how confident you feel in your ability to handle the difficulties potentially associated with the results of genetic screening". Participants responded with a score ranging from 1 ("Not capable at all") to 5 ("Fully capable"), to the following questions: "How to manage situations immediately following a genetic screening in the event of genetic risk (e.g., improving one’s lifestyle and carrying out periodic checks)", "How to manage any therapies suggested in the presence of genetic risk (e.g., hormonal or preventive therapies)" (M = 3.72, SD = 0.81, α = .810, *r* = 587).

#### Intention

Intention to undergo genetic screening was measured through a single item evaluating the urge to engage in the behavior: "In the next few months, do you intend to undergo a planned a genetic screening?". Intention was assessed according to Schwarzer (2008) [33] and Renner & Schwarzer’s (2005) indications [53] and adapted by previous applications of the HAPA model [54]. The response options were: "No, I have no intention of planning a genetic screening"; "No, but I am thinking about it, even if I am not sure"; "Yes, I am going to plan a genetic screening soon"; "Yes, I am going to plan a genetic screening and implement the preventive programs recommended by doctors".

#### Planning to undergo genetic screening

Finally, planning was assessed with modified versions of two questions used in previous literature on planning behavioral change [33, 53]. The following questions were asked: "Do you plan: (1) "When to do a genetic screening (e.g., taking work permits)"; (2) "How to do a genetic screening (e.g., whom to contact to organize)". Responses were given on a 4-point Likert scale from 1 “not true at all” to 4 “very true” (M = 1.94, SD = 0.90, α = .822, *r* = 699).

### Data analysis

Data analysis was conducted using IBM SPSS Statistics and the LAVAAN package in R statistical software [55, 56]. SPSS was employed to check the normality distribution of the variables, calculate descriptive statistics, and check for significant differences between participants with and without a family history of *BRCA1/2-related* cancer. Pearson’s correlation, point-biserial correlation, and Spearman’s rank-order correlation coefficients (ρ) were employed in a multicorrelation matrix to determine the bivariate correlations among all the measured variables. Cronbach’s alpha (α) coefficient and inter-item correlation (*r*) were calculated to evaluate the internal consistency of the constructs measured by multiple items. To assess the latent structures of multiple item constructs, the authors relied on Hair, Black, Babin, and Anderson’s (2010) [57] recommendations for identifying significant factor loadings for sample sizes less than 150 using a factor loading threshold of .45. A fully unconstrained Structural Equation Model (SEM) was conducted to test the model using the maximum likelihood with the robust standard error MLR Huber-White estimator, to overcome over-estimates due to the intention construct, measured as an ordinal item. Given the normal distribution of the constructs and intention measured with 4-response categories, MLR was the recommended estimator [58]. Hu and Bentler’s (1999) [59] guidelines for various fit indices were employed to test the SEM fit. To determine the goodness of fit, the following indexes and cut-offs were considered: the Chi-square (χ^2^) and *p*-value, the Comparative Fit Index (CFI; adequate if ≥ .90), the Root Mean Square Error of Approximation (RMSEA; adequate if ≤ 0.08) and the Standardized Root Mean Square Residual (SRMR; adequate if ≤ 0.08).

## Results

A total of 213 male participants were recruited in the study, but 88 participants did not complete the survey and thus only 125 male participants (mean age = 58.53 y/o, SD = 10.37) that completed the survey were included in the analysis. Independent Student’s t-tests and Pearson’s Chi-Square tests were performed to assess sociodemographic differences among participants (n = 125) and those who did not complete the survey (n = 88). No statistically significant differences were found by age, the presence of offspring, or current occupation. However, participants that did not complete the survey had lower educational levels [*χ*^*2*^ (5) = 13.206, *p* = .02] and a lower breast cancer risk [*χ*^*2*^ (1) = 4.822, *p* = .03]. Almost all participants were Italian (N = 123, 98.4%). Sociodemographic characteristics, including educational level and employment status, and the presence of offspring and family history of *BRCA1/2*-related cancers are presented in Table 1.

**Table 1:** Characteristics of study participants (N = 125).

Socio-demographic		Frequencies	(%)
**Age** (years), mean (sd) = 58.53 (10.37); range = 22–80			
**Education level**			
	Primary school	4	3.2
	Middle school	19	15.2
	High school	64	51.2
	University	26	20.8
	Postgraduate school	15	9.6
**Employment status**			
	Employed	68	54.4
	Unemployed	8	5.4
	Retired	42	33.6
	Other specification	7	6.6
**Offspring**			
None		39	31.2
One child		25	20
Two children		52	41.6
Three or more		9	7.2
**Family history of *BRCA 1/2* related cancer**			
Presence		64	51.2

Descriptive statistics, Pearson’s correlation, and Cronbach’s alphas are presented in Table 2. Skewness or kurtosis values proved to be in the acceptable range between -1.96 and +1.96 (SkewnessMIN = -0.842 –SkewnessMAX = 0.980; KurtosisMIN = -0.646 –KurtosisMAX = 1.237) [60].

**Table 2:** Means, standard deviations, and bivariate correlations (N = 125).

	M	SD	1	2	3	4	5	6	7	8
1. Self-efficacy	3.72	0.81	1							
2. Positive outcome expectancies	4.02	0.92	.363***	1						
3. Risk perception	3.56	1.38	.032	.181*	1					
4. Intention ^a^	-	-	.366**	.293**	.198*	1				
5. Planning	1.94	0.90	.246**	.222**	.205*	.489**	1			
6. Age	58.53	9.95	.036	-.055	-.060	.149	.010	1		
7. Offspring ^b^ = 1 (N = 88, 70.4%)	-	-	-.046	-.088	.061	.168	.129	.235**	1	
8. Family history of *BRCA1/2* cancers ^b^ = 1 (N = 64, 51.2%)	-	-	.180*	.035	.084	-.039	.011	.006	.103	1

Note: * = 0.05 level (2-tailed).

** = 0.01 level (2-tailed).

*** = 0.001 (2-tailed). Higher scores indicate higher standing on the construct (e.g.: the higher the score, the higher measured self-efficacy, positive outcome expectancies, risk perception, intention, and planning)

a = Correlations between intention and other variables are expressed with the Spearman rank-order correlation coefficient (ρ).

b = Correlations between presence of family history of *BRCA1/2* cancers (0 = absence, 1 = presence) and offspring (0 = absence, 1 = presence) and other variables are point-biserial.

**Fig 1:**
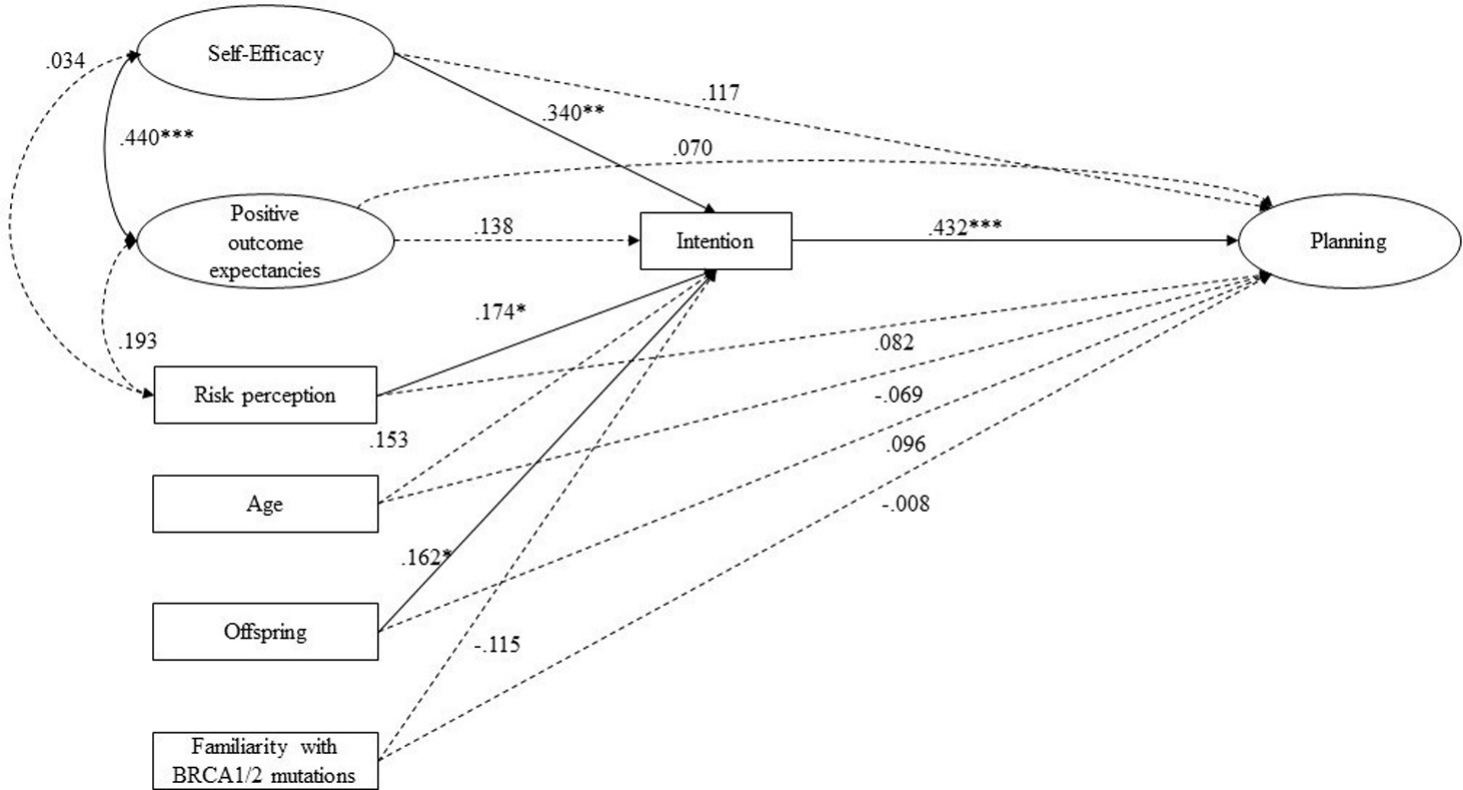
Results for the structural equation model of the HAPA: Standardized coefficients are displayed. * p ≤ 0.05; ** p ≤ 0.01; ***; p ≤ 0.001. Only significant path coefficients are displayed. Dotted lines display non-significant paths.

Bivariate correlations revealed that positive outcome expectancies are associated with self-efficacy and risk perception (Table 2). Self-efficacy and positive outcome expectancies were significantly and positively associated with intention and planning. As for the volitional phase: higher levels of intention are significantly associated with higher levels of planning. All significant correlation coefficients were below the threshold of 0.70, overcoming multicollinearity concerns [61]. Moreover, participants with a family history of *BRCA1/2* cancers presented higher levels of self-efficacy (M = 3.89, DS = 0.77, t(123) = -2.028, *p* = .05) than participants with no family history (M = 3.57, DS = 0.83). A SEM with a robust maximum likelihood standard error estimator was employed to test our hypothesized model. Participants’ age, presence of offspring, and their family history of *BRCA1/2* mutation related-cancer risk were all entered as control variables, and multi-item constructs were estimated as latent factors. Results are displayed in Fig 1.

The model exhibited a good fit with the data [χ^2^(102) = 86.971, p = .18, CFI = .981, RMSEA = .034, SRMR = .062]. All items had significant and sizeable standardized factor loadings on their multi-item constructs, ranging from .532 to .931. According to the hypothesis, the suggested distal antecedents predict the intention. Specifically, higher levels of self-efficacy (*β* = .340, *p* = .009) and risk perception (*β* = .174, *p* = .041) lead to higher levels of intention to undergo genetic screening for BRCA mutations. Thus, positive outcome expectancies showed a strong association with self-efficacy (*r* = .440, *p* ≤ .001) but did not directly affect any volitional phase outcomes (intention and planning). The unique predictor of planning was intention, showing a strong effect (β = .432, *p* ≤ .001). The presence of offspring influenced intention formation (β = .162, *p* = .044). Age and family history of *BRCA1/2* mutations risk did not influence intention or planning.

## Discussion

Pathogenic mutations in *BRCA1* and *BRCA2* are associated with an increased risk of developing breast, prostate, and pancreatic cancer, as well as other cancers, among men and women. While extensive research has been conducted on women’s specific needs and the psychological determinants of engaging in preventive behaviors, little is known about men’s intention and willingness to undergo genetic screening. In the present study, the process that leads to the intention and planning of undergoing genetic screening in men was tested using the HAPA model, controlling for age, presence of offspring to whom the potential mutation could be transmitted, and their family history of *BRCA*-related risk.

Regarding the pre-intentional phase, self-efficacy was strongly associated with intention, consistent with previous studies, highlighting that confidence in handling potential consequences plays an instrumental role. As shown in previous research on health decision making [39], and specifically preventive genetic screening [43], individuals with high self-efficacy are more prone to imagine success and anticipate potential outcomes of diverse strategies, which provides the background for considering behavioral change. Similarly, risk perception is positively associated with the development of the intention: an individual with a higher risk perception related to developing *BRCA1/2*-related cancers is more willing to undergo genetic screening. This result is consistent with previous findings on the strict connection between relative risk perception and volitional phases of behavioral change [62, 63]. In contrast, a family history of *BRCA1* and *BRCA2* genetic mutations did not play a significant role when also controlling for individual or family characteristics, and for subjective risk perception. This emphasizes the differences in transmission modalities awareness across genders within first-degree relatives [5, 8], leading to the well-known disparity in terms of knowledge of the *BRCA1/2*-related cancer risks between men and women, and consequently confirming family history as not influent [64]. The results were consistent with the theoretical framework and the literature regarding genetic screening [62, 65–67]. However, the relationship between intention (and planning) and positive outcome expectancies, age, and family history of breast-related cancer was non-significant. Developing the intention is a proximal and essential determinant for planning the concrete steps needed for enhancing preventive behavior. Intention mediates the relationship between the pre-intentional antecedents and the planning phase of behavioral change [66].

This study did not find any significant association between the volition phase and positive outcome expectancies. One explanation for this finding could be the fear of stigmatization [68–70]. The disparity between genders in studying and approaching *BRCA1/2* mutations is also reflected in social roles and discrimination [28, 71]. Cultural social roles associated with gender and communication processes are linked to the equation that *BRCA1* and *BRCA2* are «genes responsible for breast cancer» (and “only women have breasts”). Previous research also suggests a different pattern of communication between men and women on *BRCA1/2* mutations, where men are less likely than women to share thoughts on their risk, or to be included in sensitive conversations by their families [25, 28, 29]. In this specific framework, being excluded from the conversation operates together with the lack of awareness and a general sense of uncertainty related to the consequences of *BRCA1/2* mutations. Accordingly, positive outcome expectancies, operationalized as "Other people and my family members would appreciate the effort of will" or "It would increase my sense of security", may not be as self-evident in the case of *BRCA1/2* mutations as they are for other preventive behaviors (e.g.: dietary behavior or physical exercise in cardiovascular patients [54, 72], breast self-examination for women [73]).

Our findings adds impetus to the research and interventions in this field of men’s health. Our results present a complex picture characterized by a significant association between relative risk perception and the intention to undergo genetic screening for *BRCA1/2* mutations and the non-significant association between FHS-7 (i.e., an objective measure of risk) and intention. Therefore, one might propose that increasing men’s awareness regarding their vulnerability to possible *BRCA1/2* mutations, paired with specific risk evaluation and management, would consequently enhance preventive behaviors. Moreover, our results suggest risk management is significantly affected by self-efficacy.

As suggested by a recent review comparing male and female awareness management of breast cancer [74], women are targeted for awareness campaigns and undergo genetic screening and testing to a greater extent than men do [19, 25]. Specifically, they are aware of the probability of transmitting their mutations to their children, especially daughters [25]. This resonates with previous findings on women attending genetic screening: perceiving the responsibilities for the transmission of a genetic mutation, and for hereditary breast/ovarian cancers specifically, strongly affects their risk management [75, 76]. One of the most interesting results of the study highlights that men are also driven by concern for their children, regardless of family history of *BRCA1/2* related cancers and age, and the presence of children is significantly associated with the more concrete formation of intention [25, 77–79].

The present study has several limitations. First, a behavioral change model would benefit from longitudinal data. The results presented here show correlations and make no implications about causal mechanisms. Nonetheless, our results suggest the importance of exploring the applicability of the HAPA model with longitudinal and behavioral measures. Second, we selected participants based on a self-report validated measure [50], detecting the risk of *BRCA1* and *BRCA2* mutations. Family history of *BRCA1/2* related cancers and mutations should be investigated not only in terms of knowledge of relatives’ BRCA-related cancer diagnoses but also in terms of health literacy on this matter among families [80]. It is reasonable to assume that awareness of transmission mechanisms could affect the intentional dynamics and trigger pre-intentional determinants of preventive behaviors. Third, we did not assess negative outcome expectancies, which could be informative to better understand those with no intention to undergo screening within a stigmatized framework. Although Schwarzer [33] found positive expectancies sufficient to predict intention, it is reasonable to think that negative expectancies (e.g.: undergoing genetic screening for *BRCA1/2* related mutation would cause stress, nervousness) would operate in concert with self-efficacy and risk perception by balancing positive outcome expectancies. Finally, with a longitudinal design and suitable sample-size, the authors recommend operationalizing the volitional phase (e.g., intention and planning), as suggested by Renner and Schwarzer [53].

Despite these shortcomings, future interventions could benefit from these results by promoting a more thoughtful risk perception awareness and reinforce their capability of coping with the behavior and its consequences, and stress the transmission mechanisms across generations. Moreover, the present findings highlight the HAPA suitability as a theoretical and practical framework for investigating the psychological determinants of behavioral change. Intention and planning are fundamentals to enhancing health-related behavior affecting life and family dynamics. Accordingly, the positive and significant role of having children strengthens the need for family-oriented approaches to genetic counseling [69]. Finally, the present study confirms the role of volitional factors in predicting how men develop the intention and a plan for enhancing detective health behavior. Moreover, our results suggested that health campaigns should be tailored and adjusted to men’s specific demands to inform their decision-making (see [20, 81, 82]). As suggested by Pritchard (2019) [19] the terminology of the syndrome itself—*BRCA1-* and *BRCA2*-associated Hereditary Breast and Ovarian Cancer syndrome–is considered misleading and should be changed because it induces the false belief that *BRCA1/2* mutations are "women’s business" only. Further investigations are required to experimentally test the best communicative strategies to promote informed decision-making in men facing the risk of *BRCA1* and *BRCA2* mutations.

## Conclusions

Most of the information on *BRCA1* and *BRCA2* pathogenic mutations are tailored to women [74] due to the availability of effective surgical risk reduction procedures for breast and ovarian cancer among women [83–85]. Different aspects of the present findings are particularly relevant and suggest further investigation to implement balanced awareness and preventive strategies. Self-efficacy, outcome expectancies, risk perception and family history of *BRCA1/2*-related cancers play a defining role in men’s decisional process leading to genetic screening. Moreover, a change in perspective is required: men need specific attention and, consequently, the information influencing their decision-making should be tailored to their needs and perceptions. *BRCA1/2*-related mutations and cancers should not be considered and treated as a female prerogative.

## Supporting information

S1 Dataset (SAV)
